# Dietary Factors Associated with Faecal Consistency and Other Indicators of Gastrointestinal Health in the Captive Cheetah (*Acinonyx jubatus*)

**DOI:** 10.1371/journal.pone.0120903

**Published:** 2015-04-01

**Authors:** Katherine M. Whitehouse-Tedd, Sandra L. Lefebvre, Geert P. J. Janssens

**Affiliations:** 1 Cheetah Outreach, Somerset West, Western Cape, South Africa; 2 School of Animal, Rural and Environmental Sciences, Nottingham Trent University, Southwell, United Kingdom; 3 Banfield Applied Research and Knowledge Team, Banfield Pet Hospital, Portland, Oregon, United States of America; 4 Department of Nutrition, Genetics and Ethology, Ghent University, Merelbeke, Belgium; Institut Pluridisciplinaire Hubert Curien, FRANCE

## Abstract

Gastrointestinal diseases pose significant risks to captive cheetah survival and welfare. Multiple factors are thought to be associated with these diseases, but to date a comprehensive epidemiological survey of disease risk factors has not been conducted. A survey of diet and health parameters was completed for 184 captive cheetahs in 86 international facilities. Comparisons were made among dietary factors with respect to disease status and observed faecal consistency, incidence of vomiting and diarrhoea in the past 4 weeks. Extremely dry faeces were most common in cheetahs fed carcasses, but was still of low incidence (15%). Contrastingly, cheetahs fed commercially prepared diets had the highest prevalence of liquid faeces “always” or “often” (9%). Cheetahs fed raw meat diets had the highest prevalence of soft faeces with no shape (22%), as well as of firm and dry faeces (40%). No broad category of diet exerted any influence on the health parameters investigated. However, feeding of ribs at least once per week reduced the odds of diarrhoea (P = 0.020) and feeding of long bones (limbs) at least once per week was associated with a lower odds of vomiting (P = 0.008). Cheetahs fed muscle meat at least once per week had reduced odds of suffering from chronic gastritis (P = 0.005) or non-specific gastrointestinal disease (P < 0.001). The only factor identified as increasing the odds of chronic gastritis was feeding of horse “often” or “always” (P = 0.023). The findings of the current study build on existing empirical research to support a recommendation towards a greater inclusion of skeletal components. Current husbandry guidelines advocating the use of supplemented raw meat diets are likewise supported, but the use of horse meat, as well as commercially prepared diets for captive cheetahs, warrants caution until further research is conducted.

## Introduction

The cheetah (*Acinonyx jubatus*) is classified as vulnerable, with free-ranging populations threatened due to loss or degradation of their natural habitat and conflict with landowners and farmers [1]. Alongside *in situ* conservation programmes, the captive population offers a scientific resource for the improved understanding of this species’ biology, and the opportunity for these captive animals to act as educational ambassadors for their wild counterparts. Yet captive cheetahs are afflicted by a number of diseases which are rare, if at all present, in the free-ranging cheetah population, and which pose significant risks to animal survival and welfare in captivity [2].

One of these diseases, gastritis, is known to affect over 95% of the captive population in North America and South Africa [2], 55% of the European population [3], and is also known to be highly prevalent in Japan [4]. Gastritis appears as inflammation and infection of the stomach mucosa and results in debilitating gastrointestinal symptoms, including chronic diarrhoea and vomiting, weight loss and failure to thrive [5]. Gastrointestinal diseases are likely to confer significant welfare implications on affected animals and may, at least partially, explain the poor reproductive output of captive cheetahs. Chronic stress is considered to be an aetiological agent in various diseases of captivity in this species [6], and is often cited as one of the most probable causative agents to explain their high prevalence of gastritis [2,6].

Until recently, the role of diet in the gastrointestinal pathology of captive cheetahs was largely dismissed [2]. The reason cited for suggesting that diet was not a key factor in gastrointestinal disease in this species was the finding that gastrointestinal pathology was similar between captive cheetahs fed apparently divergent diets, i.e. those in North American and South African facilities [2]. However, the role of nutrition in preventing, treating and causing gastrointestinal disease is well established in domestic carnivores [7–9] and may be of equivalent significance to the captive cheetah population. This has been confirmed by the recent work of Depauw et al. [10–12], whereby a beneficial reduction in putrefactive factors (bacterial fermentation by-products) was observed in cheetahs following a dietary change from meat chunks to whole rabbit carcasses [11]. Additionally, consumption of a whole carcass diet resulted in a tendency towards improved stool consistency in captive cheetahs, compared to when the same animals were fed a raw meat diet [10]. These findings were supported by a subsequent *in vitro* study in which changes in cheetah faecal fermentation profile were elicited by animal tissue substrates (e.g. skin, fur, bone) [13]. The concept of ‘animal fibre’ (i.e. non-digestible tissues of animal origin) was thus postulated, and potential functions similar to plant fibres were suggested [10]. In 2005 an international workshop on the diseases affecting the captive cheetah population established that it was necessary to “*conduct an epidemiological analysis of risk factors for developing moderate to severe gastritis including genetic lineage*, *housing*, *diet*, *collection size and density*, *and exposure to the public*” [14]. To conduct such a comprehensive study as outlined by the workshop participants (requiring an extensive list of husbandry factors to be assessed) [14], a stepwise approach to the evaluation of potential risk factors was considered prudent in order to minimise participant burden and maximise response. Furthermore, the diagnosis of gastritis would need to be definitive, standardised and equally available to all captive animals around the world.

The clinical signs of gastritis [5] are not unique to gastritis and therefore definitive diagnosis of gastritis requires histopathological evaluation, which may not be equally available to all zoological facilities. The situation is further complicated by differing levels of veterinary vigilance, diagnostic tools and expertise associated with some zoological collections, particularly those in regions outside of the large accreditation bodies such as the Association of Zoos and Aquariums (AZA) and European Association of Zoos and Aquaria (EAZA).

As such, a more universally available method of assessing gastrointestinal health in captive cheetahs must be utilised to first establish the existence of epidemiological risk factors. Faecal consistency scoring has previously been used as a non-invasive measure of gastrointestinal health in exotic felids [15] and domestic carnivore species [16,17], and can be performed with relatively little training. Importantly, poor faecal consistency is thought to indicate gastrointestinal stress in captive carnivores [15]. Faecal consistency score has also been successfully applied in a study of tapirs (*Tapirus* spp.) by Clauss et al [18] where a significant correlation between faecal consistency and dietary energy intake in captive animals was considered indicative of inappropriate dietary factors [18].

The current study was initiated in response to the research priority identified by the AZA SSP [14], with diet selected as the primary factor to be evaluated due to the growing body of evidence warranting its investigation. A survey was designed to detect potential dietary risk factors and their relationship with faecal consistency (and/or other parameters of gastrointestinal health), and achieved this aim through the epidemiological analysis of data collected from 184 captive cheetahs housed in 86 international facilities.

## Materials and Methods

The hypothesis tested was that defined dietary factors are associated with the presence of poor faecal consistency and/or other indicators of sub-optimal gastrointestinal health in captive cheetahs. A cross-sectional epidemiological study was conducted. International facilities listed as housing cheetahs (cross-referencing between the International Species Information System (ISIS) database and the International Cheetah Studbook, as well as SSP and EEP databases) were surveyed and information gathered on the medical status of cheetahs within those collections, as well as a number of current dietary factors.

### Animals

At the time of survey, the captive cheetah population included approximately 1500 individuals, held in over 250 facilities worldwide [19]. Given that the expected prevalence of gastrointestinal illness was high (25%; 95% confidence interval (CI); 20 to 30%) among the general captive cheetah population (predicted from previous studies [2,3,20,21]), requests for participation were sent to all facilities known to house cheetahs, in order to maximise potential response. Only information on adult cheetahs (i.e. over 24 months of age at the time of survey) was collected. Pregnant and lactating females were excluded. Respondents were required to provide the animal’s international cheetah studbook number or regional studbook number, details of any medications provided to the animal in the past 4 weeks, details of any disease(s) currently suffered by the animal (as diagnosed by a veterinarian), details of the diet currently fed to the animal and details of any vitamin/mineral supplement provided. A single survey was required per animal. However, in order to reduce response-fatigue and eliminate potential bias introduced by large collections, each survey respondent was limited to a maximum of 3 surveys (i.e. 3 animals per collection). To facilitate this, respondents from large collections (>4 adult cheetahs) were asked to select 3 individuals according to their studbook number (including the animal with the lowest, median and highest studbook number within their collection).

### The survey

An online survey was conducted using a Survey Monkey Gold account (http://www.surveymonkey.com). The survey was designed to ensure ease of answering, with participants informed of the type of data required prior to initiating the online survey. Participants who did not wish to participate via the internet, or were unable to access the online survey, were provided with a PDF document of the survey and returned completed surveys by e-mail or postal service.

Respondents were identified according to e-mail or postal addresses provided in the International Cheetah Studbook, or distributed on behalf of the authors by the Cheetah SSP or EEP co-ordinators, in order to ensure distribution to the most appropriate zoo personnel. Where no address was available, the facility’s website was used to contact zoo personnel. The survey was conducted in English, with no translation provided. Each survey returned was entered into a prize draw of $1000 (USD), payable to a charity of the winning facility’s choice, in an attempt to maximise survey response rate. The survey included questions on factors listed in Table 1.

**Table 1:** Factors assessed in the global survey of captive cheetahs.

Animal factors	Dietary factors	Health factors
Age (date of birth)	Diet type	Faecal consistency^1^
Body weight (estimated or known)	Diet ingredients (specified as primary (providing >80% of the weekly intake); secondary (50–80%); or tertiary (<50%) ingredients)	Presence of any clinical signs of sub-optimal gastrointestinal health (e.g. diarrhoea and vomiting) within the past 6 months
Sex	The proportional inclusion of (i) Hide/skin, (ii), Long bones (limbs), (iii) Thoracic bones (ribs). (iv) Skulls, (v) Feet/wings, (vi) Muscle meat, (vii) Viscera, (viii) Fur/feathers, (ix) Vitamin and/or mineral supplementation used	Histopathological evidence of gastrointestinal disease**
Geographic region of housing (North America, South America, Europe, Africa, Middle East, Asia, Australasia)	Feeding frequency – daily and weekly	Presence of other systemic disease^2^
	Type of feeding schedule – fixed or random	Previous medical history relating to gastrointestinal health (> 12 months since detection of symptoms/signs)
	Meal size (kg/individual/day)	

^1^An approximate average over the past 1 month, using a 1–5 scale predefined with photo references. Faeces assessed should be exclusive of scent-marking scat, which is known to be looser, darker and more tarry in consistency than faeces voided at other times in free-ranging cheetah (Boast, L.; Durant, S.; Marnewick, K.; pers. comm.).

^2^As diagnosed by a veterinarian

### Survey design

A pilot study was conducted with 12 independent zoo professionals and results were used to refine the survey to ensure a maximum of 5 minutes response time was required. The pilot study required the questionnaire to be completed by personnel from a sub-sample of facilities; the respondents of which were subsequently interviewed in detail so as to understand their interpretation of the questions. Feedback was also obtained regarding the ease of answering the survey questions, missing or redundant categories, the time taken and any other information that respondents wished to convey. The survey was revised accordingly before being distributed to all cheetah-holding facilities.

To test whether the sick and healthy cheetahs were different in important ways other than the putative risk factors studied, the survey also included questions on variables that theoretically should not differ in distribution between sick and healthy cheetahs (i.e. diagnosis of dental disease or infection with feline immunodeficiency virus, feline leukaemia virus, calicivirus, herpesvirus, rabies virus, or *Haemobartonella*).

Researcher and participant bias was minimised by randomly selecting target cheetahs in large collections, and collecting all data regarding medical history and husbandry factors simultaneously. Respondent geographic distribution was compared to international holdings for cheetah, according to the International Cheetah Studbook [19] at the time of the survey. Completion of the finalised survey was requested of the most senior member of keeping staff that was directly responsible for the husbandry of the facility’s cheetahs.

### Faecal consistency grading

At the time of writing, no information was available to confirm the consistency of faeces associated with ‘normal’ or ‘optimal’ gastrointestinal health in cheetahs. As such, the faecal consistency scoring system was developed from the system used by veterinarians and researchers involved with companion animals [22], but utilising photographs taken of cheetah faeces (provided by an author (KWT), with the exception of one photo from a free-ranging cheetah, which was provided by K. Marnewick). Information was gathered from field researchers experienced in the collection of free-ranging cheetah scat in order to understand the range of faecal consistency exhibited by animals consuming a natural diet under free-living conditions. Field information gathered (see Table 1) suggests that free-ranging cheetah produce two types of scat; (A) normally voided faeces, and (B) faeces excreted during territory marking behaviour, with the latter being considerably looser. Faeces voided as part of territory-marking behaviour were excluded when defining the category of “normal” faecal consistency for free-ranging cheetahs. For the captive cheetahs in the present study, no distinction between types of scat was requested since the respondents were asked to “consider which [faecal score] best represents the faeces for this animal over the past 4 weeks”. Data were then collected on the frequency of each faecal score (always, often, occasionally, or never). It was assumed that territory-marking behaviour would be independent of an animal’s gastrointestinal health status (pers. obs. KWT).

The 5-point faecal scoring system developed (grades ranging from 1 to 5) included 1 point (grade 4) which was considered “normal”, and 4 points (grades 1–3; liquid, soft without shape, soft with shape and 5; extremely dry) which were considered “sub-optimal” according to free-ranging cheetah scat. The terms “normal” and “sub-optimal” faecal consistency were not recorded on the faecal scoring chart provided to the survey respondents to ensure they were effectively blinded to what could be considered abnormal. Likewise, the title of the survey was vague (“cheetah diet survey”) in order to minimise the risk of alerting the respondents to our hypothesis that an association exists between dietary factors and gastrointestinal health.

### Dietary analysis

Approximate macronutrient intake for each cheetah was estimated from data provided by respondents and calculations made using Zootrition (version 2.6, St. Louis, MO, 2006). Survey responses regarding prey animal species and animal parts fed, frequency of feeding each substance, daily amount of food intake, and frequency of feeding overall were used to estimate the nutritional content of food consumed by each cheetah. For animals fed diets consisting of a mixture of more than one ingredient, a weighting was assigned according to respondents’ assessment of the relative proportion represented by each food item. Items that were “always” fed were weighted as 100% of dietary intake, “often” as 75%, “occasionally” as 25%, “rarely” as 5% and “never” as 0%. Where respondents failed to select any option in this section, the food item was marked as 0%. These percentage weightings were only estimates of the true proportion of each food in an individual cheetah’s diet.

Percentage weightings were used to calculate the components of each cheetah's diet. With regard to the inclusion of certain animal parts in a cheetah’s diet, the assumption was made that smaller animals (i.e., guinea pigs, chickens, turkeys, rabbits, and rodents) were fed as whole or partial carcasses, and larger animals (i.e. horses, cattle, donkeys, pigs, and goats) were fed as meat on the bone (as a limb equivalent to a partial carcass) or muscle meat, with the actual format assigned according to the participant’s response to the question on diet type (carcass, raw meat, or commercially prepared diets). When survey respondents indicated some of these body parts were fed to the cheetah only once or twice/month or less frequently, those data were not included in the analysis, as these were not considered to contribute sufficiently to the animal’s typical dietary intake.

Finally, the animal’s reported total daily intake (kg) was used to estimate the proportional intake of each feed item, before the daily intake for each cheetah was entered into Zootrition and a nutritional composition report generated for each animal’s diet. Percentage weighting were used to estimate weight in grams. Since possible responses regarding the total amount of food each cheetah received were given as ranges (e.g. 1 to 1.5 kg), the midpoint of each range (e.g. 1.25 kg for a range of 1 to 1.5 kg) was used as an estimate of the total food given per day. Responses of < 1 kg/d were rounded to 1 kg, and those indicating > 3 kg/d were rounded to 3 kg.

Estimated dry matter, crude protein, crude fat, gross energy (GE), crude fibre, total dietary fibre and ash were used as putative predictor variables in statistical analyses.

### Statistical analyses

All analyses were performed with Stata software (Stata/IC 11.1 for Windows, StataCorp, Texas). For questions in which “no,” “none,” “none of the above,” or “never” was not an option, missing values were treated as an implicit “no”. In all other circumstances, they remained as missing values. Variables measured with a Likert scale were treated statistically as ordinal and were re-categorised into dichotomous variables (e.g. often or always vs occasionally or never) to facilitate interpretation.

Comparisons were made among diet types (i.e. raw meat with or without supplementation, commercially prepared, full or partial carcasses, or a mixture of these options) with respect to faecal consistency reported as observed “always” or “often” in the past 4 weeks. Other variables assessed for associations with management and health factors included current non-specific gastrointestinal disease presence (yes or no), current specific gastrointestinal diseases (e.g. acute gastritis or gastroenteritis), current non-gastrointestinal diseases (e.g. cancer or renal disease), vomiting or diarrhoea in the past 6 months, and frequency of various faecal consistencies in the past 4 weeks. Predictor variables included prophylactic treatments in the past week (e.g. vaccination or de-worming), source of current diet fed (e.g. commercially available or raw-meat based), nature of current diet fed (e.g. whole or partial carcass), types of meat fed (e.g. pork or beef or commercial [moist, semi-moist, or dry]), components of meat fed (e.g. hides, long bones, or ribs), daily amount fed (kg, as fed), feeding frequency, history of dental disease (yes or no), type of routine veterinary care provided (e.g. annual physical examination or vaccination), and individual nutrient variables estimated from the nutrition analysis. Commercial diets were defined as those available from a commercial supplier as a pre-packaged diet, whereas raw meat diets included only diets that were prepared by the housing facility as homemade recipes.

Three predictor variables were created to represent current supplements administered: nonspecific supplement (e.g. vitamins and minerals or nutraceuticals), vitamins and minerals only (yes or no), and calcium products only (yes or no). Data on cheetah age were examined for normality of distribution by construction of a histogram and performance of the Shapiro-Wilk test, which revealed a pronounced skew to the right (*P* < 0.001). Consequently, this information is reported as median and range and the values for age were logarithmically transformed to achieve a normal distribution for all subsequent analyses.

Assumptions for logistic regression were checked and univariate logistic regression was used to identify unconditional associations (odds ratios [ORs]) between outcomes and predictor variables. The χ^2^ test was used for dichotomous outcomes such as disease presence or absence, and linear regression (for ordinal frequency outcomes) were used for the sole purpose of identifying variables that would need to be controlled for in multivariate models, with the *a priori* hypothesis that multiple factors influence gastrointestinal health in cheetahs. For contingency tables in which a cell value was < 20, the Fisher exact test was used. For those in which complete data separation was not possible (i.e. total lack of outcome or predictor in 1 or more cells), the Cornfield method of OR estimation was used.

Correlations between pairs of variables were determined using the Pearson test for correlation, and one variable for each moderately correlated pair (i.e., *r* > 0.70) was selected for multivariate analyses. A liberal *P* value of 0.20 was used to identify predictor variables for inclusion in a mixed multivariate logistic regression process that allowed for controlling of clustering (similarity of cheetahs within groups) at various levels (zoo, country, or continent). Use of zoo or country as a random effect resulted in similar results but limited the study power; for this reason, continent was chosen as the only random effect. Age was included and retained as a fixed effect, regardless of *P* value, because it was strongly believed to influence the probability of disease development or history from the perspective of biological plausibility. Values of *P* < 0.05 were considered significant for results of multivariate analyses.

## Results

### Cheetahs

Completed surveys were returned for 184 cheetahs, representing 12% of the global captive cheetah population and 33% (86/260) of facilities known to house cheetahs [19,23]. Countries represented by > 6 cheetahs each included the United States of America, France, Germany, South Africa, United Kingdom, Australia, The Netherlands, Namibia, and United Arab Emirates. Countries represented by < 4 cheetahs included Austria, Belgium, India, Sweden, Italy, Spain, and Russia. Botswana, Canada, and Poland were represented by 1 cheetah each. No facility submitted surveys for > 4 cheetahs. Where incomplete questionnaires were returned, available data were used where appropriate. However, partially completed questionnaires resulted in N < 184 for some analyses.

On a regional basis, the majority of participating facilities were North American (36% of respondents) and European (34% of respondents); these regions each housed 20% of the animals listed in the International Cheetah Studbook [19] at the time of the survey. The next largest proportion of contributors was from the African region (12%), which housed slightly over one third of the listed cheetahs at the time of the survey [19]. The remaining proportion of contributors (Australasia, 7%; Asia, 6%; the Middle East and Russia, 4%) was made up of responses from regions known to house relatively small numbers of captive cheetahs [19]. No responses were obtained from facilities in Central or South America, which mirrored the small proportion of holdings in this region (0.3%; [19]).

Of the 183 cheetahs for which sex was reported, 110 (60%) were male and 73 (40%) were female. Age, which was reported for 179 cheetahs, ranged from 2.0 years to 17.5 years (median, 7.5 years). Routine veterinary care was provided in the form of physical examination with and without sedation for 56 (30%) and 25 (14%) cheetahs, respectively, and 100 (54%) received vaccinations on an annual basis (values reported are not mutually exclusive). A total of 126 (68%) received treatment as needed, whereas seven (4%) cheetahs reportedly received none of the aforementioned veterinary services, nor did they reportedly receive veterinary treatment when necessary.

### Diets

Diet type was reported for 172 cheetahs. The most common diet type fed to captive cheetahs was raw meat (n = 63 [37%]) followed by commercially prepared (35 [20%]) and carcasses (13 [8%]). A mixture of all diet types was fed to 61 (35%) cheetahs, but it was not specified whether this was a mixture of all 3 diet types, or just 2 diet types. Analysis of ingredients listed as being included in the “raw meat” diet at least once a month revealed that a carcass component of some form (either hide, skin, bone, fur, feather, feet or wing) was included in the diet of 90.5% (57/63) of the raw meat-fed cheetahs. Additionally, in raw meat-fed cheetah diets, muscle meat was included in at least every second meal for 60.3% of animals (38/63). The majority of animals fed a “mixture” diet were also fed some form of carcass component at least once a month (96.7%; 59/61). Ninety-eight (53%) of all cheetahs received dietary supplementation with products containing vitamins and minerals, calcium alone (n = 11 [6%]), plant oils (3 [2%]), or glucosamine and chondroitin sulphate (1 [1%]). Diet type varied by region (Table 2), whereby commercially prepared diets were only fed in North American facilities, and was the most popular diet type fed in this region. Carcasses were only fed in European (21%) and African (5%) facilities. Raw meat diets were common in all regions, but most popular in African facilities.

**Table 2:** Diet type fed to captive cheetahs, according to geographical location of facility (n = 86 facilities).

	Raw meat	Carcasses	Commercially prepared	Mixture
Africa	58%	5%	0%	37%
Europe	38%	21%	0%	41%
North America	21%	0%	53%	26%
Rest of the world^1^	55%	0%	0%	45%

^1^Rest of the world (includes cheetahs from Australasia, Asia, the Middle East and Russia. No cheetahs in Central or South America were surveyed).

### Faecal consistency

Faecal consistency within 1 month preceding survey completion was reported for 182 cheetahs. The most frequent faecal consistency reported as being “always” or “often” observed for captive cheetahs was “soft with shape” (51%), followed by “firm and dry” (29%). Faecal consistencies of “soft without shape” were reported under these criteria for 14% of cheetahs and of “extremely dry” for 1%.

Two (1%) cheetahs were reported as “always” having liquid faeces, 8 (4%) had liquid faeces “often”, and 46 (24%) had liquid faeces “occasionally”. Conversely, 7 (4%) had extremely dry faeces “often” and 33 (18%) had this faeces consistency type “occasionally” (Table 3).

**Table 3:** Mean faecal consistency score^1^ reported as “always” or “often” observed for animals, and prevalence of gastrointestinal disease, grouped according to diet type.

	Commercially prepared diet	Raw meat	Carcasses	Mixture of the above
Mean faecal consistency score (± SD)	3.06 (0.91)	2.77 (0.84)	3.33 (0.82)	3.16 (0.78)
Prevalence of gastrointestinal disease diagnoses (%)	11	13	8	18
Prevalence of vomiting (%)^2^	31	33	8	44
Prevalence of diarrhoea (%)^2^	34	41	8	48

^1^Faeces scored on a scale of 1–5, with 1 = liquid and 5 = extremely dry (4 = ideal or most similar to faeces observed in free-ranging cheetahs). Gastrointestinal disease reported as diagnosed by a veterinarian (presence/absence, non-specific).

^2^Within the past 6 months.

### Gastrointestinal disease

Current gastrointestinal disease was reported for 24 (13%) cheetahs with the specific nature of that diagnosis as follows (respondents could select more than 1): chronic gastritis (15), colitis (5), inflammatory bowel disease (3), parasitic enteritis (3), duodenitis (3), bacterial enteritis (2), viral enteritis (2), ulcer (2), acute gastritis (1), regurgitation (1), reflux (1), maldigestion (1), malabsorption (1), enteropathy (1), unknown (1), and other (7). Seventy-five cheetahs (41%) had diarrhoea in the past 6 months, and 65 (35%) had vomiting; 6 of 178 (3%) had intestinal parasites. The distribution of cheetahs with gastrointestinal disease by continent was as follows: Asia, 0/19; Africa, 2/22 (9%); Europe, 6/61 (10%), Australasia, 2/13 (15%); and North America, 14/65 (22%).

Current gastric reflux, acute gastritis, enteropathy, enterocolitis, maldigestion, malabsorption, cancer, drug toxicity were represented by 1 (0.5%) cheetah each. None of the cheetahs surveyed had been diagnosed with FIV infection, FeLV infection, rabies, or hemobartonellosis at any time in the past. Twelve of 175 (7%) cheetahs had been diagnosed with herpesvirus infection at some point in the past, 9 (5%) had been diagnosed with dental disease, and 4 (2%) had been diagnosed with calicivirus infection.

### Influence of diet type on faecal consistency

**Fig 1:**
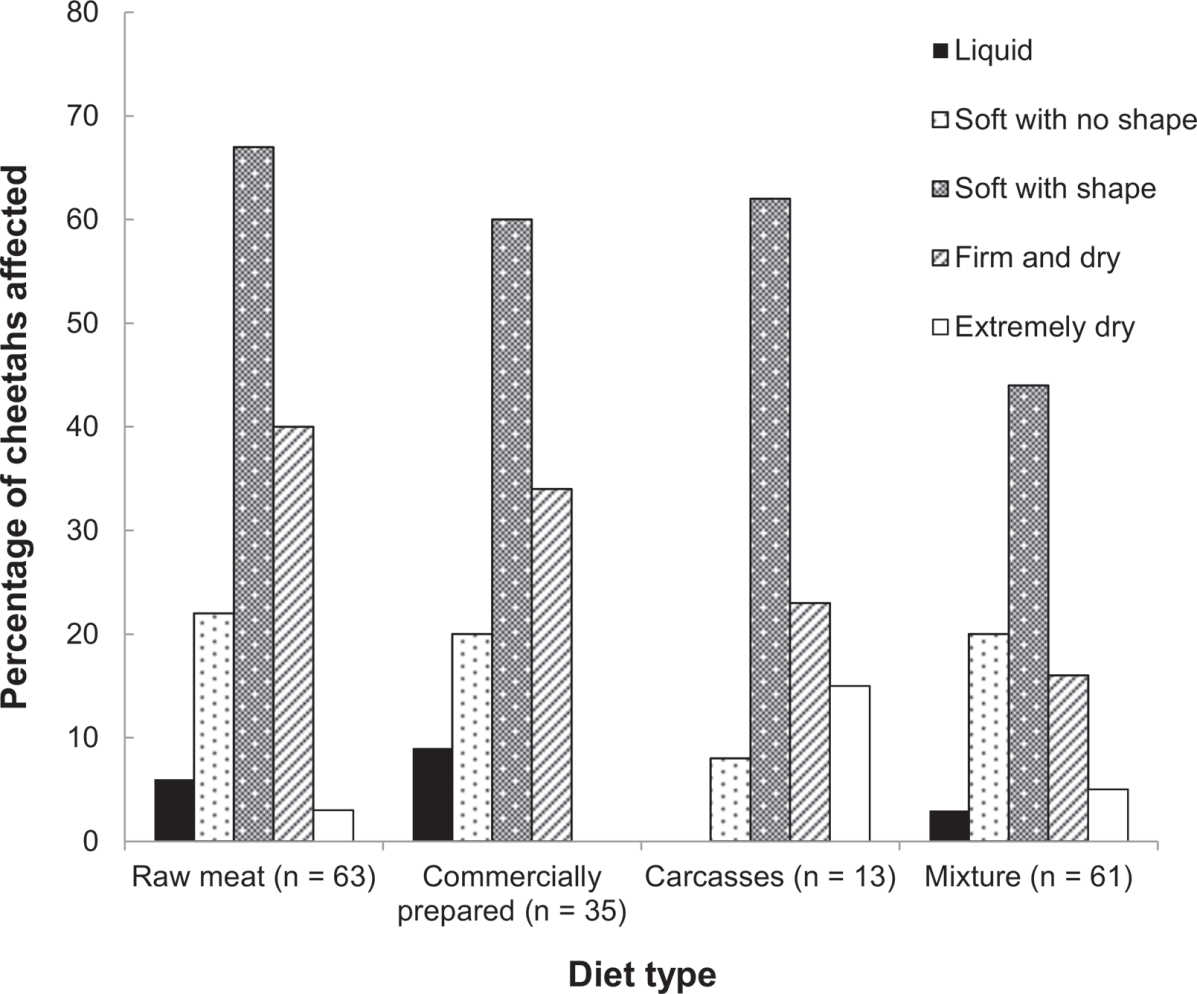
Faecal consistency within the past 4 weeks prior to completion of the survey reported for captive cheetahs, categorised according to diet type fed. Data included faecal consistency that was reported in the survey as “often” and/or “always” (values are not mutually exclusive).

Mean faecal consistency score was similar across diet types, ranging from 2.77 in raw meat-fed cheetahs to 3.33 in carcass-fed cheetahs (Table 3). The most common faecal consistency observed “always” or “often” in cheetahs over the past 4 weeks regardless of diet was “soft with shape” (Fig. 1). Extremely dry faeces were most common in cheetahs fed carcasses (2/13; 15%), and firm and dry faeces were most common in cheetahs fed raw meat (25/63; 40%). Alternatively, cheetahs fed commercially prepared diets had the highest prevalence of liquid faeces “always” or “often” (3/35; 9%), and those fed raw diets had the highest prevalence of soft faeces with no shape (14/63; 22%; Fig. 1). Cheetahs fed commercially prepared diets were significantly (*P* = 0.018) less likely to have extremely dry faeces than were those fed carcasses; extremely dry faeces was no more or less common in other diet groups. The prevalence of firm and dry faeces differed only between cheetahs fed raw versus mixed diets (*P* = 0.004), with raw meat-fed cheetahs having the greater prevalence. No other significant differences among diet types and faecal consistency were identified.

### Unconditional associations with gastrointestinal disease

Univariate analyses (without controlling for other factors simultaneously) failed to identify an association between current gastrointestinal disease and age within the cheetahs represented by the survey (OR = 1.08; 95% CI, 0.70–4.69; *P* = 0.223), nor was there an association between gastrointestinal disease and sex (OR = 0.62; 95% CI, 0.26–1.47; *P* = 0.281). Management factors identified as protective against current gastrointestinal disease were dietary supplement administration (OR = 0.36; 95% CI, 0.14–0.78; *P* = 0.022) and increasing deworming frequency (OR = 0.71; 95% CI, 0.52–0.98; *P* = 0.036), with the highest reported deworming frequency being > 3 times/year (n = 83) and the lowest frequency being none at all (13). Reclassification of the dietary supplements by type yielded no significant association for any one particular type. No particular diet type was overrepresented among cheetahs with gastrointestinal disease (Table 3); however, some associations were identified between that outcome and certain diet components. Feeding of pork on an “often” or “always” basis versus less frequently or not at all increased the odds of gastrointestinal disease 14 fold (OR, 14.45; 95% CI, 1.26–166.09; *P* = 0.032), whereas a protective effect (i.e. decreased likelihood of gastrointestinal disease) was identified for feeding of nonspecific hides (OR = 0.17; 95% CI, 0.05–0.53; *P* = 0.002), ribs (OR = 0.35; 95% CI, 0.14–0.92; *P* = 0.033), or muscle meat (OR = 0.22; 95% CI, 0.09–0.55; *P* = 0.001) at least once per week. Annual physical examination with sedation (but not without sedation) increased the likelihood of a current diagnosis of gastrointestinal disease (OR = 3.93; 95% CI, 1.62–9.53; *P* = 0.002). Diarrhoea (OR = 2.78; 95% CI, 1.15–6.74; *P* = 0.024) or vomiting (OR = 2.99; 95% CI, 1.24–7.19; *P* = 0.014) in the past 6 months also increased that likelihood. No other significant associations between factors hypothesized or not hypothesized to be associated with gastrointestinal disease were identified, with the exception of dental disease (OR = 5.84; 95% CI, 1.45–23.57; *P* = 0.013) and herpesvirus infection (OR = 5.41; 95% CI, 1.56–18.77; *P* = 0.008).

Fifteen of the 24 (63%) cheetahs with current gastrointestinal disease also had diarrhoea in the past 6 months, compared with 60 of 160 (38%) cheetahs without diagnosed gastrointestinal illness (*P* = 0.021). No association was identified between diarrhoea and age (OR = 1.04; 95% CI, 0.95–1.13; *P* = 0.113) or male sex (OR = 1.45; 95% CI, 0.79–2.67; *P* = 0.230). Diarrhoea was strongly associated with vomiting in the past 6 months (OR, 7.51; 95% CI, 3.81–14.81; *P* < 0.001) and with vaccination in the past four weeks (OR, 11.12; 95% CI, 1.34–92.36; *P* = 0.026). The prevalence of diarrhoea in the past 6 months was lowest in carcass-fed cheetahs (1/13; 8%) and was significantly higher in cheetahs fed raw meat (OR = 8.43; 95% CI, 1.09–374.01; *P* = 0.021) or mixed diets (OR = 10.88; 95% CI, 1.41–480.50; *P* = 0.008). No significant difference (*P* = 0.092) was identified between the prevalence of diarrhoea in cheetahs fed commercially prepared diets versus carcasses. Feeding of ribs at least once per week had a protective association against diarrhoea (OR = 0.47; 95% CI, 0.25–0.90; *P* = 0.023). The amount of food fed was not associated with diarrhoea, but with each 1-day increase in the frequency of feeding per week, the odds of having diarrhoea in the past 6 months increased by 40% (OR, 1.40; 95% CI, 1.03–1.94; *P* = 0.045). Contrary to the findings for gastrointestinal disease, the odds of diarrhoea increased with increasing frequency of deworming (OR = 1.30; 95% CI, 1.01–1.69; *P* = 0.049). No other significant associations with diarrhoea in the past 6 months were identified.

When the frequency of various faecal consistencies observed over the past four months was evaluated for associations with other variables, a few relationships were identified. The frequency of extremely dry faeces increased as the frequency of feeding muscle meat increased (*P* = 0.003), as well as with the increased feeding of skulls (*P* = 0.005), and partial (but not whole) carcasses (*P* < 0.001). The same association was identified between muscle meat feeding and the frequency of liquid faeces (*P* = 0.017). An increase in the amount fed was associated with an increase in the frequency of liquid faeces (P = 0.011), as was annual examination with sedation (*P* = 0.016). No other significant associations with frequency of faecal consistency were revealed through univariate analysis.

When vomiting in the last 6 months was evaluated as an outcome, 14 of the 24 (58%) cheetahs with gastrointestinal disease were identified as also having vomiting, compared with 65 of 160 (41%) cheetahs without gastrointestinal illness (*P* = 0.011). The probability of vomiting in the past 6 months increased with increasing age (values transformed to the natural logarithm; OR = 2.15; 95% CI 1.09–4.26; *P* = 0.026). Other factors associated with an increased odds of vomiting were a diagnosis of chronic gastritis (OR = 3.03; 95% CI, 1.03–8.93; *P* = 0.045) and feeding of goat “often” or “always” (OR = 3.67; 95% CI, 1.50–8.94; *P* = 0.004). As was observed for diarrhoea, the prevalence of vomiting in the past 6 months was lowest in cheetahs fed carcasses (1/13; 8%; Table 3). Cheetahs fed a mixed diet were six times as likely to have had vomiting in the past 6 months as were those fed carcasses (OR = 6.09; 95% CI, 1.23–422.08; *P* = 0.014). Feeding of long bones (OR = 0.36; 95% CI, 0.19–0.70, *P* = 0.039) or ribs (OR = 0.39; 95% CI, 0.20–0.76; *P* = 0.006) at least once per week was associated with a decreased odds of vomiting in the past 6 months. No other associations with vomiting were identified.

Males were 20% as likely to currently have chronic gastritis as diagnosed by a veterinarian as were females (OR = 0.21; 95% CI, 0.06 to 0.70; *P* = 0.006). Cheetahs with chronic gastritis were more likely than others to have vomiting in the past 6 months (OR = 3.03; 95% CI, 1.03–8.93; *P* = 0.037). Cheetahs with chronic gastritis were also more likely to have been fed horse “often” or “always” (OR = 5.13; 95% CI, 1.40–18.86; *P* = 0.007), whereas feeding of chicken “often” or “always” had a decreased odds of chronic gastritis (OR = 0.12; 95% CI, 0.02–0.94; *P* = 0.017). Other factors with protective effects against chronic gastritis were feeding of hides (OR = 0.15; 95% CI, 0.03–0.68; *P* = 0.006), muscle meat (OR = 0.20; 95% CI, 0.06–0.61 *P* = 0.002), ribs (OR = 0.14; 95% CI, 0.03–0.63; *P* = 0.003), skulls (OR = 0.12; 95% CI, 0.02–0.94; *P* = 0.043), viscera (OR = 0.53; 95% CI, 0.31–0.88; *P* = 0.015) at least once per week, as well as dietary supplementation (OR = 0.30; 95% CI, 0.10–0.88; *P* = 0.018). The only variables associated with an increased odds of chronic gastritis were annual examination with sedation (OR = 11.36; 95% CI, 3.06–42.16; *P* < 0.001) and past diagnosis of herpesvirus infection (OR = 10.93; 95 CI, 2.94–40.66; *P* < 0.001).

### Risk factors for gastrointestinal disease

Multivariate analysis revealed that, when cheetah age and continent were controlled for, feeding of muscle meat at least once per week (OR = 0.12; 95% CI, 0.05–042; *P* < 0.001) was the only variable that maintained a significant protective effect against current gastrointestinal disease. Diarrhoea in the past 6 months (OR = 4.24; 95% CI, 1.41–12.74; *P* = 0.010), annual examination with sedation (OR = 4.88; 95% CI, 1.64–14.56; *P* = 0.004), and past diagnosis of herpesvirus infection (OR = 7.01; 95% CI, 1.11–44.17; *P* = 0.038) were associated with an increased odds of current gastrointestinal disease. None of the other variables that had previously been identified as significantly associated with gastrointestinal disease, including vomiting in the past 6 months and feeding of pork, were significant.

The odds of diarrhoea in the past 6 months increased with increasing age (OR = 2.38; 95% CI, 1.06–5.31; *P* = 0.035) and with vaccination in the past four weeks (OR = 35.18; 95% CI, 3.15–392.85; *P* = 0.004). In contrast, feeding of ribs at least once per week versus less frequently reduced the odds of diarrhoea (OR = 0.40; 95% CI, 0.19–0.86; *P* = 0.020). The odds of vomiting in the past 6 months also increased with age (OR = 2.60; 95% CI 1.06–5.31; *P* = 0.015), and with feeding of goat meat “often” or “always” (OR = 3.74; 95% CI, 1.07–11.94; *P* = 0.009), as well as with current chronic gastritis (OR = 3.57; 95% CI, 1.07–11.94; *P* = 0.039). Feeding of long bones (limbs) at least once per week versus less frequently was associated with a lower odds of vomiting (OR = 0.38; 95% CI, 0.18–0.78; *P* = 0.008). Other variables identified through univariate analyses as significant were no longer significant in the multivariate analyses involving diarrhoea and vomiting as the outcomes.

In the final multivariate analysis involving chronic gastritis as the outcome, age was not significant; however male cheetahs were 76% less likely to have had chronic gastritis at the time of survey completion than were females (OR = 0.24; 95% CI, 0.07–0.85; *P* = 0.028); those fed muscle meat at least once per week were also less likely to have chronic gastritis (OR = 0.17; 95% CI, 0.05–0.59; *P* = 0.005). The only factor identified as increasing the odds of chronic gastritis was feeding of horse “often” or “always” (OR = 4.97; 95% 1.25–19.87; *P* = 0.023). The protective effect of feeding chicken “often” or “always” neared significance (OR = 0.13; 95% CI, 0.02–1.03; *P* = 0.053). Herpesvirus infection was no longer significant.

### Nutritional analysis

With age and continent controlled for, an increase in the frequency of “soft faeces with shape” was identified as associated with an increase in the grams of dry matter intake (*P* = 0.026), but not in percentage dry matter (*P* = 0.604), and with an increase in the percentage of crude fibre (*P* = 0.041). No other significant associations were identified between frequency of the various faecal consistencies and the nutrition variables established through hypothetical dietary analysis.

Controlling for age and continent, current chronic gastritis was the only dichotomous outcome evaluated for which significant associations with nutrition variables were identified. The odds of chronic gastritis increased by 1% for each 1-g increase in dry matter consumed per day (OR = 1.01; 95% CI, 1.00–1.01; *P* = 0.003), by 5% for each percentage increase in dry matter (OR = 1.05; 95% CI, 1.02–1.07; *P* < 0.001), and by 8% for each 1-g increase in protein (OR = 1.08; 95% CI, 1.02–1.14). An increase in GE (kcal/g) was associated with a considerable decrease in the likelihood of chronic gastritis (OR = 0.28; 95% CI, 0.08–0.93).

All raw survey data is available upon request to the corresponding author (KWT) although, for data confidentiality reasons, facility and staff names, as well any animal identifier that may be used to identify a facility, will be excluded.

## Discussion

The results reported here represent the largest international epidemiological survey of captive cheetahs conducted to date, representing 184 animals from 86 international facilities, in 19 countries. A range of geographic regions are represented, and the contribution of responses was regionally-biased in a manner similar to that of the relative holdings of captive cheetahs [19]. The lack of significant association detected for gastrointestinal disease and dental disease in the final model lends support to our assumption that cheetah groups (healthy versus sick) were similar except for the predictors of interest. However, the identification of past herpesvirus infection as increasing the odds of current gastrointestinal disease independent of other factors was not expected. It is therefore possible that herpesvirus status may be acting as a confounder in this study.

As expected, age was determined to be a significant risk factor for vomiting and diarrhoea, potentially indicative of known age-related increasing susceptibility to gastrointestinal disorders. However, more interestingly, female cheetahs were at greater risk of experiencing chronic gastritis than males. Previous research has demonstrated that captive females are more tense and fearful than males, which may be an adaptive trait suited to their *in situ* ecology [24]. This may result in sex-dependent differences in response to environmental stressors, and should be further investigated. In the meantime, it may be advisable to consider sex-linked differences in animal responses when reviewing management regimes aimed at minimising stress and disease risk.

Veterinary vigilance varied across facilities, with only half (49%) of surveyed animals being subject to routine health examinations, or being vaccinated annually (54%). This variability must be considered when interpreting the gastrointestinal disease data reported, as the level of veterinary vigilance is likely to influence the detection, diagnosis and treatment of gastrointestinal diseases. This is supported by the significant association identified during multivariate analysis between the conduct of annual examinations and increased likelihood of having current gastrointestinal disease and chronic gastritis. It is, hence, possible that the prevalence of gastrointestinal disease in the captive population may be underestimated in this current study as a consequence of the low examination rate for many cheetahs. It is also likely that the lower prevalence of gastritis (8%) reported in this survey, compared to pathological surveys of deceased animals (e.g. [2–4] which report 55–95% prevalence) is partially reflective of the difficulty in accurately diagnosing many gastrointestinal diseases in living animals due to the invasive nature of diagnostic techniques.

Regional differences in prevalence of gastritis were similar in the current study as with previous reports [2,3], whereby North America had a greater prevalence of gastritis than Europe, despite equivalence in number of cheetahs representing both regions, and according to regional holding data [19]. The current study also revealed a distinct regional bias in the use of carcasses or commercially prepared diet types. Raw meat feeding was shown to be the most common practice in all regions. As shown previously [25], carcass-feeding was restricted to European facilities, and a smaller proportion fed in African facilities, whereas commercially prepared diets were only fed in North American facilities. The regional bias in the use of carcass-based or commercially prepared diets for cheetahs may be due to differences in availability, cultural views on the use of certain food items, concerns regarding hygiene or storage of carcasses, or varying husbandry recommendations. Further investigation is required to elucidate whether the concomitant higher prevalence of gastritis and use of commercially prepared diets in North America has any physiological or clinical basis. Less than a third of cheetahs in the current study were reported as typically having a faecal consistency equivalent to the norm for free-ranging cheetahs (i.e.“firm and dry”). Contrastingly, nearly one quarter of all surveyed animals were reported as “occasionally” having liquid faeces, which may be explained as territory-marking related faeces, as observed in free-ranging cheetahs (L.Boast; S.Durant; K.Marnewick, pers. comm.). Nonetheless, two fifths of captive cheetahs had been observed to have diarrhoea in the past 6 months. However, the term “diarrhoea” was subject to respondent’s individual interpretation (i.e. this variable was not required to have been diagnosed by a veterinarian) and may therefore not accurately reflect a disturbed GI tract for those cheetahs. Likewise, although it must also be acknowledged that free-ranging cheetah scat may not necessarily reflect truly optimal gastrointestinal health in cheetahs, the findings of Munson et al [2] support our use of free-ranging cheetah scat as a benchmark for normality since the prevalence of disease, including gastritis, is significantly lower in free-ranging cheetahs, and animals in this population rarely exhibit the gastrointestinal lesions that are common and generally severe in captive cheetahs. Combined, these findings indicate that a noteworthy proportion of surveyed captive cheetahs may be of compromised or sub-optimal gastrointestinal health status, at least intermittently.

The “firm and dry” faecal type was most common in cheetahs fed raw meat, compared to the other diet categories, which is encouraging given the high utilisation of this diet type. The increased incidence of liquid faeces in cheetahs consuming commercially prepared diets determined here builds on the preliminary study of Lane et al [26], in which loose stools were also associated with a commercial cat food when fed to captive cheetahs. Moreover, cheetahs reported as being fed carcasses in the current study were more likely to produce “extremely dry” faeces, indicating the potential for the non-meat carcass components to act as stool bulking or firming agents. Although our epidemiological findings cannot demonstrate a causal relationship, they may most likely be explained as a response to a changed hindgut fermentation pattern in cheetahs [10,13].

Cheetahs consuming whole rabbit carcasses have previously been shown to produce faeces with lower concentrations of putrefactive compounds (e.g. indole, phenol, p-cresol), and have a lower incidence of diarrhoea, compared to when the same animals were fed a raw meat diet [10]. The mechanism behind the effect of diet format on faecal consistency is yet to be elucidated, although it has been hypothesised that undigested animal tissue, such as bone, hair, skin and cartilage, present in carcass diets may have a beneficial impact on the gastrointestinal ecosystem, including influencing motility, absorption, gastric emptying and microbial fermentation processes [10,13]. The latter has been confirmed *in vitro* such that animal fibres increased the production of short-chain fatty acids by cheetah faecal inocula [13].

In terms of gastrointestinal disease, it had previously been suggested that diet was unlikely to play an important role in gastrointestinal disease in captive cheetahs [2]. The argument was made that since cheetahs in South African facilities suffered from a high incidence of gastritis, despite consuming a diet similar to that of free-ranging cheetahs (which exhibited little gastrointestinal pathology), diet was therefore unlikely to be aetiological [2,6]. However, these authors did not conduct a dietary survey and their assumption about the popularity of carcass component inclusion in the diet of cheetahs held in South African facilities has been largely refuted by the current study.

Gastritis as well as non-specific gastrointestinal disease risk was significantly decreased when muscle meat was fed at least once a week. Additionally, links were identified between carcass-feeding and decreased diarrhoea and gastritis prevalence. However, since carcass components may be less likely to be fed to cheetahs with a history of diarrhoea, it is feasible that the low prevalence of diarrhoea seen in carcass-fed animals is misleading. Nonetheless, it is unlikely that the protective effect detected for muscle meat resulted arbitrarily from a pre-existing decreased utilisation of muscle meat for cheetahs with symptoms of gastrointestinal disease. Typical veterinary advice includes increasing dietary digestibility in animals with compromised gastrointestinal health [8], and therefore the relatively increased digestibility of muscle meat would render it more likely to be increased, rather than decreased, in the diet of ill animals. Moreover, a protective effect against vomiting and diarrhoea was identified for the feeding of specific carcass components (ribs and long bones) during multivariate analysis, and supports the low prevalence of diarrhoea in carcass-fed cheetahs. As such, it is likely that the protective effect of muscle meat as well as some carcass components (ribs and long bones) determined here is supportive of a more naturalistic feeding regime, as suggested by Marker et al [27]. However, when diet was evaluated by the general categories of raw meat, commercial or carcass-based meals, no diet type was identified as having a significant effect on concurrent gastrointestinal disease status. The small sample size of diseased animals is likely to have reduced our power to detect any association using these broad categories, and these findings should not be used to rule out further investigation.

Since the putrefactive compounds associated with poor faecal consistency are known to be linked to a range of chronic intestinal inflammatory disease in domestic carnivores [28], concern was raised previously regarding the poor faecal consistency associated with diets low in animal fibre [10]. Subsequently, it has been demonstrated that faecal biomarkers of inflammation were increased in cheetahs fed a raw meat diet, compared to when fed carcasses [12]. These biomarkers are secreted during acute intestinal inflammation [29–31] and it is therefore feasible that the epidemiological association between feeding carcass components and decreased odds of vomiting and diarrhoea (potentially reflecting undiagnosed gastrointestinal disease) is driven by the presence of animal fibre in such diets. Previously, exotic felids (excluding cheetahs) receiving either commercial diets or raw meat were reported to have a higher prevalence of non-specific health problems (57% and 50%, respectively) compared to carcass-fed animals (39%; [32]). These findings are supported in the current study, whereby, the prevalence of diarrhoea and vomiting was lowest in carcass-fed cheetahs, and no carcass-fed animals were reported as having been diagnosed with gastrointestinal disease. Likewise, cheetahs fed commercially prepared diets had the highest prevalence of gastrointestinal disease and vomiting, and the second highest prevalence of diarrhoea. In contrast to our findings, Lane et al [26], suggested that a commercially prepared diet offered some beneficial effect against gastritis in cheetahs [26]. However, no significant difference in rate of gastritis development or gastritis scores were actually detected, and the commercial diet provided by Lane et al [26] was not fed exclusively (it was supplemented with chicken carcasses in order to improve faecal consistency to an acceptable level [26]). This corresponds directly with both the liquid faeces reported in cheetahs fed commercially prepared diets and the protective effect for chicken determined in our study. Surprisingly, Lane et al [26] suggested that a commercial cat food was not detrimental to captive cheetah health when their study was not (by their admission) designed to determine this, and would not meet even basic requirements for assessment of nutritional adequacy in companion animals [33]. Nutritional assessment of commercial and raw meat diets fed to cheetahs have revealed significant deficiencies and potential toxicities [11,34,35], and require the evaluation of nutrient composition as well as serum biochemistry. As such, neither the results of our study, nor those of Lane et al [26], should be interpreted as providing evidence of diet suitability for the maintenance of captive cheetah health without further testing.

With that stipulation in mind, two specific meat types were identified as risk factors for gastrointestinal disease in the current study. The feeding of either horse meat or goat meat on an “often” or “always” basis resulted in a stark increase in gastritis and vomiting risk, respectively. Subsequently, one of the nutrient risk factors identified for chronic gastritis was increased crude protein intake. These findings appear mutually supportive since goat and horse meat are relatively high in protein compared to other meat types fed (Table 4; data for beef, chicken, lamb and turkey obtained from the NRC [36]; for goat from Webb et al [37] and for horse from Badiani et al [38]).

**Table 4:** Nutrient composition of various meat products.

	Dry matter (%)	Crude protein (% DM)	Crude fat (%DM)
Beef (meat)^1^	40.6	15.0	23.5
Chicken (meat + skin)^1^	38.2	17.6	20.3
Lamb (meat)^1^	40.5	16.6	23.4
Turkey (meat)^1^	30.9	13.3	16.0
Goat (meat)^2^	34.6	23.4	11.5
Horse (meat)^3^	29.1	19.8	6.63

^1^ National Research Council [36]

^2^ Webb et al [37]

^3^ Badiani et al [38]

Increased protein content may act directly in the gastrointestinal tract to stimulate bacterial ammonia production, modify microbiota composition and increase putrefactive fermentation by-products, with detrimental consequences for gastrointestinal health [8,10,13,39]. However, since crude protein is anticipated to be high in all carnivorous diets, including that of free-ranging cheetahs, the digestibility and subsequent hindgut fermentation pattern of the protein is more likely to be the contributory factor, rather than crude protein content *per se*, in any association with gastrointestinal disease. Crude protein digestibility of horse based diets in exotic felids has been shown to be greater than that of beef-based diets [40], although goat meat has not yet been investigated. Furthermore, since horse (in particular) is typically fed as muscle-meat alone (without ingestible bones and cartilage), its relative lack of “animal fibre” may further increase its risk of yielding higher concentrations of putrefactants in the hindgut of carnivores [10,12].

Our finding of a protective effect for muscle meat (regardless of prey species) against gastrointestinal disease is consistent with the observation that “firm and dry” faeces were most common in cheetahs fed a raw meat diet, but slightly at odds with our understanding of the beneficial role of animal fibre, of which muscle meat contains only relatively moderate quantities (e.g. collagen). In our analysis of a “raw meat” diet, this was the term used to define the animal’s usual (entire) diet over the past month, whereas “muscle meat” was one of multiple ingredient types that were assessed on their frequency of inclusion in the animal’s diet. As such, further analysis of dietary ingredients used revealed that the “raw meat” diet typically included skeletal components (i.e. a meat-on-the-bone diet or with additional carcass components provided), and that the “muscle meat” component was not fed in isolation, but was fed in combination with additional sources of animal fibre.

The association between gastrointestinal problems and dry matter (DM) intake, which was also found to be associated with increased odds of producing soft, shapeless faeces, may indicate a response to particularly large meals, as has been reported in free-ranging wolves [41]. Since digestibility tends to decrease with increasing feed intake, potentially due to an increased transit time [42], it is feasible that the association between DM intake and looser stools reflects an increased presence of undigested material entering the hindgut, thereby increasing the substrate available for microbial fermentation and putrefactant production. This in turn may explain the increased risk of gastritis with greater DM intake. In contrast, a beneficial effect was observed for increased GE intake. However, this may actually reflect the compositional differences between carcasses and commercially prepared diets. The higher carbohydrate and lower fat content of commercially prepared diets typically results in lower GE, compared to carcass meals, whereby this nutrient effect may be driven by diet type rather than energy intake alone.

Due to the cross-sectional design of this study, the temporal nature of any associations identified could not be determined. The small number of cheetahs with current gastrointestinal disease (n = 24) may have limited the power of any multivariate model for detecting significant associations with other variables, particularly when the other variables were represented by small numbers of cheetahs as well. As with all epidemiological studies, this research provides evidence of potential risk factors, but cannot provide empirical evidence of a causal relationship between husbandry factors and indicators of gastrointestinal health or disease in captive cheetahs. However, our findings highlight risk factors which are likely to require further study.

Nonetheless, the immediate benefit to the zoological community of this research is two-fold. Firstly, the greater understanding of the potential dietary drivers of faecal consistency and gastrointestinal health in captive cheetahs provides zoos with the opportunity to better evaluate, monitor and improve this important parameter in captive animal health. Secondly, the development and circulation of a standardised faecal scoring system (available upon request from the corresponding author), based on evidence from free-ranging and captive cheetahs, will enable a global-standard, by which facilities can measure their cheetah’s apparent gastrointestinal health and set a benchmark for which to aim.

## Conclusions

An epidemiological relationship between diet and gastrointestinal disease indicators has been demonstrated and significant risk factors requiring further investigation and empirical testing were revealed. The use of muscle meat, with or without carcass components, was associated with an improved gastrointestinal health status, in contrast to the use of commercially prepared diets. Vomiting and diarrhoea were less likely to occur in animals fed carcass components (ribs and long-bones), while muscle meat-fed cheetahs were at reduced risk of gastritis and non-specific gastrointestinal disease, as well as having improved faecal consistency. Prevalence of gastrointestinal disease was lowest in carcass-fed animals although no epidemiological relationship could be detected for any broad diet category during multivariate analysis. Interestingly, specific prey species were identified as having a significant impact on risk of gastrointestinal disease during univariate analysis, whereby chicken was found to have a protective effect (reducing the odds of gastrointestinal disease), but horse meat and goat were shown to increase the risk of gastritis, or vomiting (respectively). Horse was the only prey species to maintain a significant (detrimental) relationship with gastritis risk during multivariate analysis, which may be related to its protein content and/or digestibility, or its relative lack of animal fibre. The findings of the current study builds on existing empirical research and zoo husbandry guidelines to support a recommendation towards a greater inclusion of carcass and muscle meat components in the diet of captive cheetahs.
